# Health needs and access of homeless people

**DOI:** 10.1590/0034-7167-2024-0462

**Published:** 2026-03-16

**Authors:** Tiago Rocha Pinto, Beatriz Kaori Ianaba, Joice Vitória de Oliveira Palma, Larissa Yasmin da Silva Marques, Carolina Siqueira Mendonça

**Affiliations:** I Universidade Estadual Paulista. Botucatu, São Paulo, Brazil

**Keywords:** Homeless Population, Needs and Demands for Health Services, Access to Health Technology, Unified Health System, Qualitative Research., Población en Situación de Calle, Necesidades y Demandas de Servicios de Salud, Acceso a la Tecnología en Salud, Sistema Único de Salud, Investigación Cualitativa.

## Abstract

**Objectives:**

to understand the homeless population’s health needs and to characterize their experiences with accessing the Health Care Network.

**Methods:**

a qualitative, descriptive, and exploratory study, conducted with homeless individuals who use a reception center in a medium-sized municipality in the countryside of São Paulo. Twelve semi-structured interviews were conducted and analyzed using the historical-cultural approach and Vygotsky’s explanatory method.

**Results:**

the information gathered supported the development of three core topics of meaning and sense: The experience of living on the streets; Major health problems and concerns; Health care and access.

**Final Considerations:**

the Brazilian Health System is able to be active in the homeless population’s lives, but the complexity of the phenomenon points to the need for coordinated attention and care actions that go beyond the health sector.

## RESUMEN

**Objetivos:**

comprender las necesidades de salud de la población en situación de calle y caracterizar sus experiencias de acceso a la Red de Atención de Salud.

**Métodos:**

investigación cualitativa, descriptiva y exploratoria realizada con personas en situación de calle que utilizan un albergue en un municipio de tamaño mediano del interior de São Paulo. Se realizaron y analizaron doce entrevistas semiestructuradas mediante el enfoque histórico-cultural y el método explicativo de Vygotsky.

**Resultados:**

la información apoyó el desarrollo de tres significados y sentidos fundamentales: La experiencia de vivir en la calle; Los principales problemas y preocupaciones de salud; El acceso a la salud y la atención médica.

**Consideraciones Finales:**

el Sistema Único de Salud puede intervenir activamente en la vida de las personas en situación de calle, pero la complejidad del fenómeno resalta la necesidad de acciones coordinadas de atención y cuidado que trasciendan el ámbito sanitario.

## INTRODUCTION

Defined as a heterogeneous population group, which has in common extreme poverty, interrupted or weakened family ties and the lack of regular conventional housing, the homeless population (HP) in Brazil has been intensifying in recent decades far beyond the large metropolises^(1)^.

Data from the Institute of Applied Economic Research estimated that the number of homeless people in the country exceeded 281,000 in 2022, following the COVID-19 pandemic, accounting for a 38% increase since 2019. The study warns that the increase in homeless people is much greater in proportion compared to the general population (211% and 11%, respectively) for the period between 2011 and 2021, which corresponds to 1 in every 1,000 people in Brazil living on the streets^(2)^.

The plurality of experiences of HP highlights vulnerabilities, such as food insecurity, unavailability or difficulty in accessing drinking water, sleep and affection deprivation, in addition to exposure to various etiological vectors for various diseases and injuries^(3,4)^. Likewise, it is also possible to observe the predominance of sexually transmitted infections, severe malnutrition, oral problems, psychological suffering, use and abuse of legal and illegal drugs, physical and sexual violence, pregnancy, and chronic diseases without periodic control^(5)^. Aspects related to living on the streets that make the illness process of this population more complex^(6)^.

The report by the Brazilian National Health Council and the Brazilian National Human Rights Council, published in 2021, denounced the increase in the number of HP, as well as the vulnerabilities that were intensified during the COVID-19 pandemic, with greater risks of contagion and worsening of conditions^(7)^. In this context, it is worth highlighting that the federal government’s decision-making and omissions contributed to worsening the impacts of COVID-19 and resulted in massive deaths that could and should have been avoided. Mortality among homeless people was five to ten times higher than in the general population^(8)^.

Furthermore, this political scenario and the related social project led to a worsening of the country’s economic and social situation, which impacted the population’s daily lives and led to a significant increase in homeless people. At this time, these were working-class people who could no longer pay their rent and bills and took to the streets to prioritize the search for food^(9,10)^.

Although the country has seen significant progress since the establishment of the Brazilian National Policy for the Homeless Population in 2009, there are numerous challenges for its implementation and effectiveness throughout the national territory^(11)^. Due to the high vulnerability and the various health conditions prevalent in this population, it is important to highlight that reports of refusal to go to health units due to episodes of poor care in hospitals, denial of care and impediment to entry into health units are still recurrent^(12)^.

In this regard, research that reveals the experiences of access and care in health services, experienced by HP, can contribute to its implementation.

## OBJECTIVES

To understand the HP’s health needs and characterize their experiences of accessing health services in a medium-sized municipality in the countryside of São Paulo.

## METHODS

### Ethical aspects

The study was submitted to and approved by the Research Ethics Committee of the School of Medicine of Botucatu, as recommended by the National Health Council Resolution 466/2012, meeting all the criteria and care for conducting research involving human beings^(13)^. In order to preserve informant confidentiality and anonymity, the representation “I1 to I12” was adopted to name each of the speeches.

### Theoretical-methodological framework

The theoretical-conceptual framework used was the historical-cultural approach based on Vygotsky’s explanatory method^(14,15)^. With the “word with meaning” as the unit of analysis, it was possible to structure the basis for the creation of the so-called “discourse meaning cores”, in a movement that sought to identify central topics and issues reported by individuals, understood more as those that generate motivation, emotion and involvement than by their frequency in the report^(16,17)^.

### Study design

This is a qualitative, exploratory, case study conducted with homeless people who use the “Welcoming Space” (*Espaço Acolhedor*) in the municipality of Botucatu, in the countryside of the state of São Paulo. The choice of qualitative research was due to its unique ability to investigate representations, beliefs, values, explanations, meanings, and significance, as well as to understand the context in greater depth from participants’ perspective^(18)^. To maintain methodological rigor, the COnsolidated criteria for REporting Qualitative research protocol was used as a support tool for research development.

### Methodological procedures

It followed all the historical-cultural approach precepts based on the analysis of cores of meaning and sense.

### Study setting

The study was conducted at the “Milton Francisco de Oliveira” Welcoming Space, a 24-hour municipal service linked to the Department of Social Assistance designed to temporarily accommodate up to 25 adult users of both sexes. For people in transit, the service offers shelter for up to five business days, and for residents, the stay can be extended for up to six months. This service provides food, basic personal hygiene and clothing cleaning, access to civil documentation, referrals for job training and health care, as well as support for family reintegration, provision of an institutional address for reference, and other needs identified by professionals^(19)^.

Botucatu, in turn, is a municipality of around 150,000 inhabitants, located 250 km from the capital, in the south-central region of the state of São Paulo, which is connected by the Marechal Rondon and Castelo Branco highways^(20)^. Currently, the city, with a Human Development Index of 0.800, stands out for its scientific reputation, including its School of Medicine and regional *Hospital das Clínicas*, as well as its national and international health care standing. In 2021, during the COVID-19 pandemic, the municipality carried out an effectiveness study of the Oxford/AstraZeneca vaccine, distributed in Brazil by the *Fundação Oswaldo Cruz*.

The decision to contact users of the “Welcoming Space” was made for convenience and took into account the opportunity to more easily access this population. All users present at the time of the interviews, without cognitive impairments, and over 18 years of age who agreed to participate in the study by signing the Informed Consent Form were invited to participate. Those who were not present at the site at the time of collection, as well as those who refused to participate for any reason, were excluded.

### Data source

Twelve homeless people were interviewed from September 2023 to January 2024, with theoretical saturation of information being used as the criterion for suspending data collection at the 12^th^ interview. Data collection was obtained through semi-structured interviews conducted in person in a noise-free location, jointly defined with the homeless person and the Welcoming Space, and in compliance with confidentiality guidelines. The interviews used a script composed of openand closed-ended questions. The open-ended questions addressed the interviewees’ life stories, their experience living on the streets, including during the pandemic, their illnesses, and their experiences accessing health and care services. The closed-ended questions addressed sociodemographic information. It is worth noting that the script used was reviewed and adapted after the pilot interview. Similarly, the pilot interviews were not included in the sample for analysis.

### Data collection and organization

To collect data, the researchers visited the site on different days and during peak times, specifically during lunch and dinner hours. All users present on these days and times were invited to participate in the interviews. No user was excluded due to age or other impairments that prevented them from understanding and responding to the interview questions. It is worth noting that no women were found to be invited to participate in the study at the time of data collection. The interviews lasted an average of 30 minutes and were recorded and transcribed verbatim for later analysis. The choice of semi-structured interviews stems from the possibility of deepening, comprehensiveness, and diversity in the understanding process based on a script that guides the conversation, but also allows for dialogic flexibility to accommodate new topics and questions raised by interviewees^(18)^.

### Data analysis

The statements’ analytical-interpretative process followed a material organization process based on a cursory material reading and organization, which allowed for the organization of the so-called “pre-indicators” that formed a broad framework of possibilities for constructing the nuclei. A second reading allowed for a process of agglutination of pre-indicators, whether by similarity, complementarity, or contrast^(16,17)^. The next stage was the analysis itself, which began with an intra-nucleus process and progressed to an inter-nucleus articulation. This process involved grasping the determinations, motivations, and needs that constituted these forms of meaning, thus enabling the recognition of production of meanings, their contradictions, and their relationships with the social, cultural, political, and economic context. The very construction and the name given to each nucleus constituted an analytical movement, given that they bring together and highlight issues relevant to understanding the aspects researched^(16,17)^.

## RESULTS

Concerning the profile, all interviewees were male, between the ages of 24 and 64, with the most predominant groups being between 40 and 48 (33.33%), and 62 and 64 (33.33%), with an average age of 49.16 years. In relation to skin color, there was a predominance of individuals who identified as white (58.33%), followed by brown (33.33%) and black (8.34%). Regarding marital status, the majority were single (75%), with a minority of interviewees being divorced (25%). The majority of participants were Catholic (50%), followed by Evangelicals (33.33%) and those who declared no religion (16.67%). Aa for education, the majority did not complete elementary school (50%), followed by those who had incomplete high school (41.67%) and completed higher education (8.33%). In relation to health problems, high blood pressure was the most prevalent among participants (25%), but other diseases were also reported, such as diabetes mellitus, tuberculosis, and hepatitis C. Regarding the length of homelessness, there was a predominance of periods of less than one year (41.67%), followed by six years (33.33%), two years (16.67%), and three years (8.33%).

Similarly, we also observed that our interviewees mentioned family problems, unemployment, alcoholism, and drug addiction as the main factors that led to and maintained their status as homeless people. We also recognized the occupation of recyclable waste collector as the most prominent work activity among HP. The “Welcoming Space” also stands out as an important support and assistance device for most of interviewees.

### The experience of living on the streets

The first core of meaning presents elements that address the experience of living on the streets and the various meanings attributed to it. Most participants in this study described living on the streets as a difficult experience, permeated by suffering and hardship.

Climate-related aspects stand out, such as exposure to cold and rain, and even difficulty sleeping due to the dangers and risks they face daily.

*The worst part is being in the cold when it rains.* (I1)

*Cold* [...] *and I don’t have a blanket or a change of clothes. Yesterday I slept in the bathroom at Brasil Japão* [Square] *because it was so cold.* (I11)

*Very dangerous* [...] *sleeping with one eye open and the other closed, it’s sad and cold* [...] *during the rainy season you have to keep moving from place to place, terrible and very dangerous.* (I2)

*Sleeping is the biggest challenge on the street.* (I3)

*Many people are dying.* (I10)

Added to this are situations of prejudice, discrimination and unnecessary violence on the part of the police, as well as reactions of shock and sadness on the part of family members who are unhappy at recognizing them in this condition.

*I’ve been beaten by the police. I’ve been discriminated against for using drugs when I don’t. People say you’re using when you don’t. I’ve been beaten, I’ve been left in the middle of the woods by a police officer who wanted to kill me, because he thought I did something when I didn’t. It’s very humiliating* [...] *I’ve been called a thief, a bum, shameless, you name it.* (I12)

*When I was actually on the street, I didn’t even see what I was doing. I was recycling, you know, I was going through the city’s trash. One day, my daughter saw me there and cried.* (I5)

During the COVID-19 pandemic, the experience of living on the streets was not specifically defined and/or considered a moment of increased risk and/or vulnerability. There was little mention of negative experiences, as most interviewees were not homeless during this period. Only two contracted COVID-19, which was associated with a negative experience due to isolation.

*It was really bad. I caught COVID twice. My mother almost killed me. I was isolated here. The first time, I ran away. I had no television, nothing. Then it was seven days, and I ran away. The doctor said it was seven days, and they said I had to stay for ten.* (I5)

*I caught COVID, I stayed at the gym, I was there for 14 days. God forbid, so much suffering.* (I6)

### Major health problems and concerns

In the formation of this second nucleus, statements are gathered that illustrate not only the main health demands and needs, but also the different meanings in the way they relate to professionals and teams in the face of their concerns.

In their entirety, the interlocutors of this study expressed the use and/or abuse of alcohol and other drugs as being something that accompanies them daily in their lives on the streets.

*Yes, it’s hard to live on the streets without alcohol, just for survival.* (I2)

*I never liked marijuana. I only used it once, but I didn’t like it. Damn it when I used cocaine. It was like marriage, you know, love at first sight, the same thing.* (I12)

*I drink a bottle of cachaça* [Brazilian beverage] *a day.* (I1)

*I used cocaine for ten years and crack for the last two years.* (I8)

In this context, there are also those who are constantly fighting to avoid falling into chemical dependency and alcoholism again.

*I’ve used alcohol, cocaine, and crack, but not right now.* (I5)

*All of them, with alcohol, and then I switched to crack. I haven’t used drugs in eight years. I’ve never liked marijuana.* (I6)

*Yes, several. I haven’t used alcohol in three months.* (I1)

*No, no chemicals, just marijuana.* (I10)

As a result of and/or aggravated by alcohol and drug consumption, a series of other health problems are present and follow HP’s daily lives.

*I’ve had a heart attack, but it was because of drinking, two cardiac arrests.* (I1)

*I had a lot of seizures because I only drank and didn’t eat. I went two, three days without eating* [...] *a woman came by and called an ambulance for me, and they took me to the emergency room, then they took me to the ER, then they took me to the nursing home, and I’ve been there for seven months. And I have cataracts.* (I2)

*When I was younger, I had a stroke. I fainted and almost died. They told me I couldn’t smoke, drink, or anything, and I do all of that. So, I don’t think I’m too worried.* (I8)

Likewise, we also recognize reports that carry the marks of violence on their bodies.

*I was stabbed and had surgery.* (I3)

*I don’t have a disease; I just have a bullet lodged in my lung.* (I10)

### Access and health care

This core concept encompasses interviewees’ perceptions of access to and care received at different levels of health care. Concerning the health problems identified, we recognized that the Brazilian Health System (In Portuguese, *Sistema Único de Saúde* - SUS) is present in the lives of all interviewees, especially through the basic and primary care offered by the municipality’s Basic Health Units.

*I go to the clinic because it’s really close to where I work, right, Cohab I, where my sister worked. I get great service; the staff there is incredibly nice. I only go when I’m feeling really bad.* (I1)

*I feel good. I always take a little chocolate for Carlos, a chubby guy who works as a security guard at the clinic I usually go to. I haven’t had any negative experiences with the service here in Botucatu.* (I2)

*I went to the Cohab I clinic, and I always got good service. Just a while ago, I needed a dentist; I went there, and they had one. I don’t think there’s much to complain about when it comes to health care here in Botucatu. They’re very welcoming, they’re very nice with the CRAS, the basic food basket, and the gas.* (I8)

Similarly, the importance and role of a Street Outreach Office (SOO) for HP is highlighted, proving to be an important psychosocial care device due to its active search and mediation work with the municipality’s Health Care Network.

*The only medicine I take is for seizures. The girls from the street clinic pick up the medicine and bring it here.* (I1)

*I use the street clinic, the health center, and the girls here at the shelter.* (I3)

*I take double the medicine. The Street Outreach Office comes here, right? So, I talk to them if something’s wrong.* (I5)

*The first thing I do is call the head of the Street Outreach Office, and then she sends the whole team to see me. If things don’t go well, then it’s the emergency room* [with tuberculosis]. *It all came together, I couldn’t tell. A dry cough that bothered me. It didn’t take long; the girls came right away.* (I9)

Regarding their assessment of access and care received in different services and levels of health care, positive feelings predominated, highlighting situations of care, empathy, and commitment of professionals and teams towards HP, especially when it comes to Primary Health Care.

*I receive excellent care, even when I’m homeless. The girls come there every day to check my blood pressure.* (I1)

*I received excellent care. I went because I was weak, I wasn’t eating, and I went to get medication, which I still take today.* (I4)

*At UNESP, I receive excellent care. When I call an ambulance, it arrives quickly; it doesn’t take five minutes.* (I1)

*Always good, I’ve always been well treated and well looked after. Very good, and the times I needed it, they saved my life. Because of the stabbing, I spent three months in the ICU.* (I3)

However, it was also possible to ascertain, among the plurality of meanings, expressions of discontent regarding the treatment received in the municipality’s emergency services.

*It was good here. At the ER, it wasn’t good. They don’t value people who live on the streets, they really don’t. It’s ridiculous how they treat us.* (I5)

*In the emergency room, they’re incredibly disrespectful. I sat there and they didn’t see me, and they even called the police because I was drunk. I went through the emergency room. I spoke to the social worker, I went to the doctor, I went back there to get the medication. It took over an hour, and then I freaked out, they called the police, and I walked out and they beat me.* (I6)

*No, no. In the emergency room, I had it. In the emergency room, there are rude people too, it takes a while, not all of them, right? Let’s separate things. There are some rude receptionists there, but it’s fine. I heard, “Yeah, it’s like this every day here, but when I had a few drinks, we’d go every ten to two weeks, but it’s fine”.* (I12)

## DISCUSSION

Participant sociodemographic characteristics are consistent with the HP recognized by the Single Registry (In Portuguese, *Cadastro Único* - CadÚnico) in the country, identified as mostly male (87%), adults (55% between 30 and 49 years old) and black (68%, 51% of whom are brown and 17% black), and with some type of disability (15%). The majority can read and write (90%), and have had a formal job (68%). The main way to earn money was by working as a recyclable waste collector (17%), and among the main reasons given for finding themselves in this situation were family problems (44%), followed by unemployment (39%) and alcoholism and/or drug use (29%). They reported sleeping on the streets (55%), reaching 70% in the North region. In the southeast, the largest proportion of people sleep in shelters is found (41%), with the majority not living with their families on the streets (92%) and rarely having contact with relatives outside of the homeless (61%)^(1)^.

Concerning HP’s health, the last national survey carried out in 2008 already indicated that 29.7% of respondents stated that they had some health problem, the most relevant being hypertension (10.1%), followed by psychiatric problems (6.1%), HIV/AIDS (5.1%) and vision problems/blindness (4.6%), and 18.7% said they used some medication^(21)^.

In this study, all interviewees were male, and no women were found at the site during the interviews. Despite the lack of information regarding the number of male and female users assisted by the site, the results are similar to other studies in the literature and to the CadÚnico database. Regarding education, a large proportion of HPs do not complete elementary school^(22)^. However, regarding skin color, this study differs from the data contained in the literature, since the majority of participants consider themselves white. Concerning skin color, it is worth noting that, according to information from the last demographic census, the municipality of Botucatu has a predominantly white population (69.6%), with only 5.5% declaring themselves black and 24% brown^(19)^.

As for the experience of living on the streets, HP faces several adversities in their daily lives, generating great physical and emotional overload. Thus, “it is by walking that one knows the street, and it is in the process of knowing it that one learns where to walk”^(23)^, and from this, a way of existence is constructed, with a greater predisposition to illness on the streets^(24)^.

Concerning sleep, HP sleeps more during the day due to the increased movement in cities, giving them greater security at that time^(25)^. Nighttime is generally a period of greater risk exposure for HP, accompanied by the use of psychoactive substances and low temperatures that intensify during the early morning hours, making the night a period of permanent alert^(25)^. In another study, HP reported being accustomed to this situation^(26)^, a similar narrative from one of the interviewees in this study. This may be associated with the freedom that the street gives them, being able to use drugs and alcohol without being judged by their families.

Feelings such as distrust, fear, and indifference are most often portrayed by HP, reinforcing social invisibility^(22)^. The “expulsion technologies” and “anti-homeless architecture” that society creates increasingly materialize the stigmatization and discrimination of this population^(27)^.

Regarding the COVID-19 pandemic, social inequality creates distinct risks of illness. The need for isolation can create barriers to shelter admission due to the demand for space and materials, and the continuous 14-day stay. Furthermore, there is a conflictual relationship between HP and the shelters due to strict rules regarding the use of alcohol and drugs in these facilities, the prohibition of families and couples staying together, and the prohibition of pets^(6,22)^.

In relation to major health problems and concerns, the literature indicates that drug use and variables related to instability and family breakdown are the main reasons this population ended up on the streets, aspects also present in the HP of this study. Several authors highlight the same difficulties faced by the interviewees, also adding unemployment as a cause^(26)^. Romantic or family breakdown can trigger feelings of disillusionment or even depression, and may be closely linked to alcohol and drug use^(22)^.

Furthermore, cultural and sociopolitical environments influence the use and abuse of legal and illegal drugs^(28)^. On the streets, drugs become an element of communication and social interaction, bringing together individuals with common problems faced in this environment^(29)^. Added to this is the cost of drugs such as crack or *cachaça*, which is less than the cost of a meal^(6)^. This drives the use of these products, in addition to masking hunger^(23)^ and the cold, serving as a means to relax and sleep^(30)^.

Situations of violence, use of alcohol and other drugs, and lack of monitoring/treatment of chronic conditions are the main factors for seeking health services^(26)^. Regarding health problems in this population, the most prevalent are chronic non-communicable diseases, substance abuse, and sexually transmitted infections, which could also be seen in the research with the presence of individuals with high blood pressure, diabetes mellitus, tuberculosis, and hepatitis C^(25)^.

As for health access and care for participants in this study, the results differ from the findings of the last nationwide survey, which found that, regarding access to health care, 43.8% of respondents stated that they first seek out the hospital/emergency room when they are sick, and 27.4% seek out a primary care health care unit^(21)^.

SOOs address various health problems and needs specific to this population through shared actions integrated with Primary Care, formed by multidisciplinary teams^(24)^. However, due to the various obstacles that are placed on this population, they tend to be afraid and distrustful of a professional approach, hence the importance of building bonds and organizing teams so that these individuals have reference professionals^(25)^.

The SOO is also responsible for carrying out HP registration, with the SUS card being the most valued document by this population, as it is made and delivered immediately, without the need for proof of address or presentation of pre-existing documents^(25)^.

The articulation of the health network is necessary, as it enables joint actions directed at HP, in order to provide expanded care for these individuals and identify their needs, since, for this population, the territory of life is broad, dynamic and mobile^(24,31)^.

Scientific literature also reveals this dichotomy regarding health care services, with delays being the most frequently reported problem, yet they are always addressed. The services most sought by HP are emergency services, because this population does not typically visit health care services regularly, making this communication only in emergencies^(26)^.

Services are not prepared to address this population’s peculiarities, as there is still a lot of prejudice and discrimination among health professionals themselves^(26)^. The lack of knowledge among professionals and society about the life experiences of people with HP reinforces this population’s vulnerabilities and exclusion, contradicting the constitutional charter and the SUS principles^(32)^.

In this context, the profile of interviewees stands out: people living on the streets but who were at the time of the research in a shelter and care institution: Welcoming Space. Welcoming Space users commit to a set of rules, such as personal hygiene and other practices, in addition to relying on the support of the institution’s professionals and intersectoral coordination. These aspects differentiate them from people who are actually living on the streets and favor access to health care, suggesting a bias and limitation in the research.

### Study limitations

It is understood that the findings presented here do not exhaust the complexity of the phenomenon under analysis. Further studies and analyses on the topic are necessary to continue advancing our understanding of the various determinants that permeate and condition the issue. Furthermore, although the study did not plan to select gender-specific participants, only males participated in the interviews. This limitation was due to the lack of women present at the time of data collection.

### Contributions to health, nursing, or public policy

It is recognized that the work developed revealed specificities and particularities about the phenomenon under analysis, enabling us to broaden our understanding of the topic and its implications for daily care in nursing and other areas.

## FINAL CONSIDERATIONS

The qualitative design allowed for an understanding of interviewees’ social reality and their human productions, both particular and constructed in the world of relationships and representations-immeasurable aspects. Furthermore, this design, which aligns with the national curricular guidelines for medical school in the training of generalist, humanistic, critical, and reflective medical professionals, represents an alternative to the positivist model and is based on dialogue with the humanities and natural sciences, allowing student researchers to engage in dialogue with interviewees’ humanities, relationships, and intersubjectivity.

In general, we found that the information obtained in this study corroborates the findings of national and international literature on the subject, especially with regard to HP sociodemographic characteristics, with the exception of the skin color marker, in which the majority of our interlocutors defined themselves as white men.

The analysis employed through Vygotsky’s explanatory method allowed us to understand the phenomenon in a particularized way and linked to local idiosyncrasies, with emphasis on Basic Health Units and the team at the municipality’s SOO, recognized as the main agents of this population’s health demands and needs.

The context of the COVID-19 pandemic did not significantly change these individuals’ daily lives, but it is possible to infer that its effects and consequences also impacted the fact that they found themselves and remained homeless, highlighting the complexity and need for intersectoral and interprofessional actions with them.

We recognized the limitations of research with HP developed at the local level, but we highlighted the importance of further studies on their experiences of accessing health services, in order to contribute to increasing the visibility of this population’s main health demands and needs, as well as in the production of information that supports the development and implementation of programs, public policies and decision-making in similar contexts.

## AVAILABILITY OF DATA AND MATERIAL

The research data are available only upon request.
