# Supplementary material for: Health needs and access of homeless people
Source: Rev Bras Enferm. 2026 Mar 16;79:e20240462. doi: 10.1590/0034-7167-2024-0462 (PMC12991434; doi:10.1590/0034-7167-2024-0462)
Supplement: Translation

# Translation: Necessidades e acesso à saúde de pessoas em situação de rua

**DOI:** 10.1590/0034-7167-2024-0462pt

**Authors:** Tiago Rocha Pinto, Beatriz Kaori Ianaba, Joice Vitória de Oliveira Palma, Larissa Yasmin da Silva Marques, Carolina Siqueira Mendonça

**Affiliations:** I Universidade Estadual Paulista. Botucatu, São Paulo, Brasil

**Keywords:** População em Situação de Rua, Necessidades e Demandas de Serviços de Saúde, Acesso à, Tecnologia em Saúde, Sistema Único de Saúde, Pesquisa Qualitativa.

## Abstract

**Objetivos:**

compreender as necessidades de saúde da população em situação de rua e caracterizar as experiências de acesso à Rede de Atenção à Saúde.

**Métodos:**

pesquisa qualitativa, descritiva e exploratória, desenvolvida com população em situação de rua que utilizam uma unidade de acolhimento em um município de médio porte no interior de São Paulo. Foram realizadas 12 entrevistas semiestruturadas, analisadas a partir dos referenciais da abordagem histórico-cultural e do método explicativo de Vigotski.

**Resultados:**

as informações subsidiaram a elaboração de três núcleos de significação e de sentidos: A experiência de viver nas ruas; Os principais problemas e preocupações com a saúde; O acesso e cuidado com a saúde.

**Considerações Finais:**

o Sistema Único de Saúde consegue se fazer atuante na vida das populações em situação de rua, mas a complexidade do fenômeno aponta para a necessidade de ações coordenadas de atenção e cuidado que vão além do setor da saúde.

## INTRODUÇÃO

Definida como um grupo populacional heterogêneo, que possui em comum a pobreza extrema, os vínculos familiares interrompidos ou fragilizados e a inexistência de moradia convencional regular, a população em situação de rua (PSR) no Brasil vem se intensificando nas últimas décadas muito além das grandes metrópoles^(1)^.

Dados do Instituto de Pesquisa Econômica Aplicada estimaram que a PSR no país superou as 281 mil pessoas em 2022 após a pandemia de COVID-19, representando um aumento de 38% desde 2019. O estudo alerta que o aumento de PSR é muito maior em proporção, comparado à população em geral (211% e 11%, respectivamente) para o período entre 2011 e 2021, o que corresponde a 1 em cada 1.000 pessoas no Brasil vivendo em situação de rua^(2)^.

A pluralidade de experiências de PSR evidencia vulnerabilidades, como insegurança alimentar, indisponibilidade ou dificuldade de acesso à água potável, privação de sono e falta de afeto, além da exposição a diversos vetores etiológicos para várias doenças e agravos^(3,4)^. Do mesmo modo, também é possível observar o predomínio de infecções sexualmente transmissíveis, desnutrição severa, problemas bucais, sofrimento psíquico, uso e abuso de drogas lícitas e ilícitas, violência física e sexual, gravidez, e doenças crônicas sem o controle periódico^(5)^. Aspectos relacionados à vivência nas ruas tornam mais complexo o processo de adoecimento dessa população^(6)^.

O relatório do Conselho Nacional de Saúde e do Conselho Nacional de Direitos Humanos, publicado em 2021, denunciou o aumento do número de PSR, assim como das vulnerabilidades que foram intensificadas no contexto da pandemia de COVID-19, com maiores riscos de contágio e agravamento dos quadros^(7)^. Nesse contexto, destaca-se que a tomada de decisão e omissões por parte do governo federal contribuíram para tornar ainda pior os impactos da COVID-19, e resultaram em mortes massivas que poderiam e deveriam ter sido evitadas. Com relação à PSR, a mortalidade foi de cinco a dez vezes maior, quando comparada à população geral^(8)^.

Além disso, esse cenário político e o projeto de sociedade relacionado levaram ao agravamento da situação econômica e social no país, que impactou o cotidiano de vida da população e o aumento significativo da PSR. Nesse momento, tratavam-se de pessoas pertencentes à classe trabalhadora que não conseguiam mais pagar seus aluguéis e contas e foram às ruas priorizando a busca por alimento^(9,10)^.

Embora o país tenha acompanhado significativos avanços a partir da instituição da Política Nacional da População em Situação de Rua em 2009, são inúmeros os desafios para sua implantação e efetivação em todo território nacional^(11)^. Em detrimento da alta vulnerabilidade e dos diversos adoecimentos à saúde prevalentes nessa população, é importante ressaltar que ainda são recorrentes os relatos de recusa em ir para unidades de saúde devido a episódios de mau atendimento em hospitais, negação de atendimento e impedimento de entrada nas unidades de saúde^(12)^.

Nesse sentido, pesquisas que revelem as experiências de acesso e cuidado em serviços de saúde vivenciadas pela PSR podem contribuir para sua implantação.

## OBJETIVOS

Compreender as necessidades de saúde da PSR e caracterizar as experiências de acesso a serviços de saúde em município de médio porte do interior paulista.

## MÉTODOS

### Aspectos éticos

O estudo foi submetido e aprovado pelo Comitê de Ética em Pesquisa da Faculdade de Medicina de Botucatu, conforme preconizado pela Resolução do Conselho Nacional de Saúde nº 466/2012, atendendo a todos os critérios e cuidados para a realização de pesquisas envolvendo seres humanos^(13)^. No intuito de preservar o sigilo e anonimato dos informantes, foi adotada a representação “E1 a E12” para nomear cada um dos discursos.

### Referencial teórico-metodológico

O marco teórico-conceitual utilizado foi a abordagem histórico-cultural com base no método explicativo de Vigotski^(14,15)^. Tendo a “palavra com significado” como unidade de análise, foi possível estruturar a base para criação dos chamados “núcleos de significação do discurso”, em um movimento que buscou captar temas e questões centrais relatadas pelos indivíduos, entendidas mais como aquelas que geram motivação, emoção e envolvimento do que por sua frequência no relato^(16,17)^.

### Tipo de estudo

Pesquisa de natureza qualitativa, exploratória, do tipo estudo de caso, desenvolvida com PSR que utiliza o “Espaço Acolhedor” no município de Botucatu, no interior do estado de São Paulo. A opção pela pesquisa qualitativa se deu por sua particularidade em permitir a investigação de representações, crenças, valores, explicações, sentidos e significados, e a compreensão de contexto, de modo mais aprofundado, a partir do ponto de vista dos participantes^(18)^. Para manter o rigor metodológico, foi utilizado o protocolo *COnsolidated criteria for REporting Qualitative research* como ferramenta de apoio para o desenvolvimento da pesquisa.

### Procedimentos metodológicos

Seguiram-se todos os preceitos da abordagem histórico-cultural a partir da análise em núcleos de significação e de sentido.

### Cenário do estudo

O estudo foi desenvolvido no Espaço Acolhedor “Milton Francisco de Oliveira”, serviço municipal de funcionamento ininterrupto 24 horas vinculado ao Departamento de Assistência Social, destinado a acolher em caráter temporário até 25 usuários adultos de ambos os sexos. Para pessoas em trânsito, o serviço oferece acolhimento por até cinco dias úteis, e para munícipes, a permanência pode se estender por até seis meses. Esse serviço oferece alimentação, cuidados básicos de higiene pessoal e higienização de vestimentas, acesso à documentação civil, encaminhamento para capacitação para o mercado de trabalho, para assistência à saúde, além de suporte para reintegração familiar, oferecimento de endereço institucional para referência e outras necessidades detectadas pelos profissionais^(19)^.

Botucatu, por sua vez, é um município de cerca de 150.000 habitantes, localizado a 250 km da capital, estando situado na região Centro-Sul do estado de São Paulo, que se interliga pelas rodovias Marechal Rondon e Castelo Branco^(20)^. Atualmente, a cidade com Índice de Desenvolvimento Humano de 0,800 se destaca pela referência científica da Faculdade de Medicina e pela referência regional do Hospital das Clínicas, além de nacional e internacional no cenário da saúde. Em 2021, durante a pandemia de COVID-19, o município realizou estudo de efetividade da vacina Oxford/AstraZeneca, distribuída, no Brasil, pela Fundação Oswaldo Cruz.

A opção pelo contato com usuários do “Espaço Acolhedor” foi por conveniência, levando em consideração a oportunidade de acessar mais facilmente esta população. Foram convidados a participar todos os usuários presentas no local no momento das entrevistas, sem impedimentos cognitivos e maiores de 18 anos que aceitassem participar da pesquisa, através da assinatura do Termo de Consentimento Livre e Esclarecido. Foram excluídos aqueles que não foram encontrados no equipamento no momento da coleta, bem como os que se recusaram participar por qualquer motivo.

### Fonte de dados

Foram ouvidas 12 PSRs no período de setembro de 2023 a janeiro de 2024, adotando como critério a saturação teórica das informações para suspensão da coleta na 12ª entrevista. As informações foram obtidas por meio de entrevistas semiestruturadas realizadas de forma presencial em local livre de ruídos, definido conjuntamente com PSR e Espaço Acolhedor, e que respeitou as diretrizes de confidencialidade das informações, a partir de roteiro composto por perguntas abertas e fechadas. As perguntas abertas versavam sobre a história de vida dos entrevistados, sua experiência de viver nas ruas, inclusive durante a pandemia, suas enfermidades, e suas experiências de acesso aos serviços de saúde e de cuidado. As perguntas fechadas, por sua vez, tratavam de informações sociodemográficas. Vale destacar que o roteiro utilizado passou por revisão e adequação após entrevista piloto. Do mesmo modo, as entrevistas piloto não compuseram a amostra para análise.

### Coleta e organização dos dados

Para a coleta de dados, as pesquisadoras compareceram ao local em diferentes dias e nos momentos de maior circulação de usuários, a saber os horários de almoço e janta. Todos os usuários presentes nesses dias e horários foram convidados a participar das entrevistas. Nenhum usuário foi excluído por questões de idade ou comprometimentos que impossibilitassem a compreensão e resposta às perguntas da entrevista. Vale destacar, na ocasião das coletas de dados, que não foram encontradas mulheres a serem convidadas para participação no estudo. As entrevistas tiveram duração média de 30 minutos, sendo gravadas e transcritas na íntegra para posterior análise. A opção por entrevistas semiestruturadas decorre da possibilidade de aprofundamento, abrangência e diversidade no processo de compreensão a partir de um roteiro que oriente a interlocução, mas também que permita a flexibilidade dialógica, de modo a absorver novos temas e questões trazidas pelos entrevistados^(18)^.

### Análise dos dados

A trajetória analítico-interpretativa dos depoimentos obedeceu a uma organização do material a partir da leitura flutuante e organização do material, o que permitiu organizar os chamados “pré-indicadores”, que compuseram um quadro amplo de possibilidades para construção dos núcleos. Uma segunda leitura permitiu um processo de aglutinação dos pré-indicadores, seja pela similaridade, pela complementaridade ou pela contraposição^(16,17)^. O passo seguinte foi a sua análise propriamente dita, que se iniciou por um processo intranúcleo, avançando para uma articulação internúcleos. Tal processo compreendeu a apreensão das determinações, motivações e necessidades que constituíram tais formas de significar, para, com isso, poder se reconhecer a produção dos sentidos, suas contradições e relações com o contexto social, cultural, político e econômico. A própria construção e o nome dado a cada núcleo já constituíram um movimento de análise, tendo em vista que reúnem e evidenciam questões relevantes para compreensão dos aspectos pesquisados^(16,17)^.

## RESULTADOS

Em relação ao perfil, todos os entrevistados são do sexo masculino, na faixa etária de 24 e 64 anos de idade, sendo mais predominantes as faixas entre 40 e 48 anos (33,33%), e 62 e 64 anos (33,33%), tendo 49,16 anos em média. Com relação à cor da pele, houve uma predominância de indivíduos que se identificam como brancos (58,33%), seguidos de pardos (33,33%) e pretos (8,34%). Acerca do estado civil, a maior parte é solteiro (75%), sendo a minoria dos entrevistados divorciada (25%). A maioria dos participantes era católica (50%), seguida de evangélicos (33,33%) e aqueles que declararam não ter religião (16,67%). No que se refere à escolaridade, a maior parte não completou o ensino fundamental (50%), seguida dos que tiveram ensino médio incompleto (41,67%) e superior completo (8,33%). A respeito dos problemas de saúde, a hipertensão arterial é a mais prevalente entre os participantes (25%), no entanto outras doenças também foram relatadas, como a diabetes mellitus, tuberculose e hepatite C. Quanto ao tempo de situação de rua, houve uma predominância de períodos menores de um ano (41,67%), seguidos de seis anos (33,33%), dois anos (16,67%) e três anos (8,33%).

De modo semelhante, também pudemos constatar em nossos interlocutores a menção aos problemas familiares, desemprego, alcoolismo e dependência química como os principais fatores que os levaram e os mantêm na condição de PSR. Também reconhecemos a ocupação de catador de lixo reciclável como a atividade de trabalho que se destaca entre a PSR. Ressalta-se ainda o “Espaço Acolhedor” como importante dispositivo de suporte e apoio para a maioria dos entrevistados.

### A experiência de viver nas ruas

No primeiro núcleo de significação, são trazidos elementos que versam sobre a experiência de viver nas ruas e dos diversos sentidos a ela atribuídos. A maioria dos participantes deste estudo descreveu a vivência nas ruas como sendo algo ruim e permeada por sofrimentos e dificuldades.

Destacam-se aspectos relacionados ao clima, como a exposição ao frio e à chuva, até à dificuldade de dormir diante de perigos e riscos a que estão submetidos diuturnamente.

*A parte pior é ficar no frio quando chove.* (E1)

*Fria... e eu não tenho uma coberta e uma troca de roupa. Ontem eu dormi no banheiro da* [praça] *Brasil Japão de tão frio que tava.* (E11)

*Muito perigoso* […] *dorme com um olho aberto e outro fechado, é triste e frio* […] *época de chuva tinha que ficar mudando de lugar em lugar, terrível e muito perigoso.* (E2)

*Dormir é a maior dificuldade na rua.* (E3)

*Muita gente morrendo.* (E10)

Somam-se a isso situações de preconceito, discriminação e violência desnecessária por parte da polícia, bem como reações de espanto e tristeza por parte de familiares inconformados por lhes reconhecer nessa condição.

*Eu já apanhei de polícia. Já fui discriminado por usar droga e não usar. A pessoa fala que você tá usando e não usa. Já apanhei, já fui largado no meio do mato por policial que queria matar, por ele achar que eu fiz e eu não fiz uma coisa. É muito humilhante* […] *já me chamaram de ladrão, vagabundo, sem vergonha, de tudo qualquer nome.* (E12)

*Quando eu tava na rua mesmo, eu nem via o que eu fazia. Eu fazia reciclagem, né, ficava mexendo nos lixos da cidade. Um dia, minha filha me viu lá e chorou.* (E5)

Durante a pandemia de COVID-19, a experiência de viver nas ruas não foi significada de modo particularizado e/ou enquanto um momento de maior risco e/ou vulnerabilidade. Houve pouca menção a experiências ruins, pois a maioria dos entrevistados não se encontrava em situação de rua neste período, sendo que apenas dois pegaram COVID associada como vivência negativa diante do isolamento.

*Foi bem ruim, peguei duas vezes COVID. Minha mãe quase matou eu. Fiquei isolado aqui. Na primeira vez, eu fugi, não tinha televisão, nada, aí deu sete dias, eu fugi. A médica falou que era sete dias e eles falaram que tinha que ficar dez.* (E5)

*Eu peguei COVID, fiquei no ginásio, fiquei 14 dias lá. Deus me livre, muito sofrimento.* (E6)

### Os principais problemas e preocupações com a saúde

Na conformação desse segundo núcleo, estão reunidas falas que ilustram não apenas as principais demandas e necessidades de saúde, mas, também, os diferentes sentidos no modo com que se relacionam com profissionais e equipes diante de suas preocupações.

Em sua totalidade, os interlocutores deste estudo manifestaram o uso e/ou abuso de álcool e outras drogas como sendo algo que lhes acompanha cotidianamente em suas vidas nas ruas.

*Sim, é difícil conviver na rua sem o álcool por questão de sobrevivência mesmo.* (E2)

*Maconha nunca gostei, usei uma vez só, mas não gostei. Maldito quando eu usei a tal da cocaína. Foi a mesma coisa que um casamento, sabe, amor à primeira vista, mesma coisa.* (E12)

*Eu bebo uma garrafa de cachaça por dia.* (E1)

*Usei cocaína por dez anos e crack nesses últimos dois anos.* (E8)

Nesse contexto, também estão aqueles que travam uma luta constante para não se ver imerso na dependência química e no alcoolismo novamente.

*Já fiz uso de álcool, cocaína, crack, mas no momento não.* (E5)

*Todas elas, com álcool e depois fui para o crack. Faz oito anos que eu não uso droga. Nunca gostei de maconha.* (E6)

*Já, várias. Álcool faz três meses que eu não uso.* (E1)

*Não, química nenhuma, só maconha.* (E10)

Em decorrência e/ou agravadas pelo consumo de álcool e drogas, uma série de outras problemáticas de saúde se faz presente e acompanha a vida diária da PSR.

*Eu já tive infarto, mas foi por causa da bebida, duas paradas cardíacas.* (E1)

*Eu tive muita convulsão, pois eu só bebia e não comia, ficava dois, três dias sem comer* […] *passou uma moça e chamou a ambulância pra mim e me levaram para o PS, depois me levaram para o SARAD, depois me trouxeram para o espaço acolhedor e estou aqui por sete meses. E tenho catarata.* (E2)

*Quando eu era mais novo, eu tive começo de AVC. Desmaiei e quase morri. Falaram que eu não podia fumar, beber e nem nada, e eu faço tudo isso. Então, acho que não me preocupo muito.* (E8)

Do mesmo modo, também reconhecemos relatos que carregam as marcas da violência em seus corpos.

*Levei uma facada e fiz cirurgia.* (E3)

*Doença não tenho não, só tenho uma bala alojada no pulmão.* (E10)

### Acesso e cuidado à saúde

Nesse núcleo, repousam as concepções dos entrevistados quanto ao acesso e cuidados recebidos nos diferentes níveis de atenção à saúde. Em relação aos problemas de saúde identificados, pudemos reconhecer que o Sistema Único de Saúde (SUS) se faz presente na vida de todos os entrevistados, especialmente por meio dos cuidados básicos e primários ofertados pelas Unidas Básicas de Saúde do município.

*Eu vou no posto, porque fica pertinho de onde eu fico lá, né, da Cohab I onde minha irmã trabalhava. Sou muito bem atendido, o pessoal de lá é bom demais. Eu só vou quando eu estou muito mal.* (E1)

*Me sinto bem. Sempre levo um chocolatinho pro Carlos, um gordão que é vigia no postinho que tenho costume de passar. Não tive experiência negativa no atendimento aqui em Botucatu.* (E2)

*Eu ia no posto da Cohab I, sempre era bem atendido. Esses tempos atrás mesmo eu precisei de dentista; fui lá e eles tinha. Acho que, na parte da saúde aqui de Botucatu, não tem muito o que reclamar, não. São muito gente boa a parte de acolhimento, são bem bacana a parte do CRAS, a parte da cesta básica, do gás.* (E8)

De modo semelhante, ressaltam-se a importância e o papel do Consultório na Rua (CnaR) junto à PSR, revelando-se enquanto um importante dispositivo de atenção psicossocial pelo seu trabalho de busca ativa e mediação com a Rede de Atenção à Saúde do município.

*Único remédio que eu tomo é para convulsão. As meninas do consultório da rua que pegam o remédio e trazem aqui.* (E1)

*Uso o consultório da rua, posto de saúde e as meninas aqui do espaço acolhedor.* (E3)

*Tomo remédio dobrado. O Consultório na Rua vem aqui, né, aí eu converso com eles se alguma coisa estiver dando errado.* (E5)

*A primeira coisa é chamar a chefe do Consultório na Rua, e aí ela manda a equipe inteira para me ver. Se não tiver bom, aí é pronto-socorro* [com a tuberculose]. *Veio tudo junto, não deu pra distinguir não. Uma tosse meio seca que me incomodava. Nem deu muito tempo não, as meninas já vieram.* (E9)

Em relação à avaliação que fazem do acesso e do atendimento recebidos nos diferentes serviços e níveis de atenção à saúde, predominaram os sentidos positivos que ressaltam situações de cuidado, empatia e compromisso dos profissionais e equipes para com a PSR, em especial, quando se tratam dos cuidados primários à saúde.

*Sou muito bem atendido, mesmo quando eu estou na rua. As meninas vão lá todo dia medir minha pressão.* (E1)

*Fui bem atendido. Fui por causa de fraqueza, não estava se alimentando e fui pegar remédio, e tomo até hoje.* (E4)

*Na UNESP lá, sou bem tratado. Quando chamo a ambulância, chega rapidinho; não demora cinco minutos.* (E1)

*Sempre boa, sempre fui bem tratado e atendido bem. Muito bom, as vezes que precisei, salvaram a minha vida. Por causa da facada, fiquei na UTI três meses.* (E3)

Todavia, também foi possível averiguar, entre a pluralidade de sentidos, manifestações de descontentamento quanto ao tratamento recebido em serviços de urgência e emergência do município.

*Aqui foi bom. No PSA, não foi bom, não. Gente que mora na rua eles não dão valor para gente não, não dão valor mesmo, ridículo como eles tratam a gente.* (E5)

*No pronto-socorro, eles têm uma falta de respeito do caramba. Ficar sentado e não me atenderam, e ainda chamaram a GCM ainda, porque eu estava alcoolizado, né, passei pelo atendimento lá no PS. Falei com a assistente social, passei pelo médico, voltei lá para darem o medicamento, demorou mais de uma hora, aí eu surtei já, chamaram a GCM, aí eu sai andando e eles batendo em mim.* (E6)

*Não, não. No pronto-socorro, eu tive, no pronto-socorro tem gente sem educação também, demora, nem todos, né. Vamos separar as coisas, tem umas recepcionistas ali sem educação, mas está bom. Eu ouvia: “É, está todo dia aqui é que quando eu tomava umas pingas a gente ia a cada dez a 15 dias, mas está bom”.* (E12)

## DISCUSSÃO

As características sociodemográficas dos participantes se coadunam com a PSR reconhecida pelo Cadastro Único (CadÚnico) no país, identificados como majoritariamente do sexo masculino (87%), adultos (55% entre 30 e 49 anos) e negros (68%, sendo 51% pardas e 17% pretas), e com algum tipo de deficiência (15%). A maioria sabe ler e escrever (90%), e já teve emprego com carteira assinada (68%). A principal forma para ganhar dinheiro foi no trabalho como catador de lixo reciclável (17%), e entre os principais motivos apontados para se encontrar nessa situação, estão problemas familiares (44%), seguidos de desemprego (39%) e alcoolismo e/ou uso de drogas (29%). Informaram que dormem na rua (55%), chegando a 70% na região Norte. No sudeste, encontra-se a mais expressiva proporção de pessoas que dormem em albergues (41%), sendo que a maior parte não vive com suas famílias na rua (92%) e dificilmente tem contato com parentes fora da condição de rua (61%)^(1)^.

No que se refere à saúde da PSR, a última pesquisa nacional realizada em 2008 já apontava que 29,7% dos entrevistados afirmavam ter algum problema de saúde, sendo os mais relevantes a hipertensão (10,1%), seguida de problemas psiquiátricos (6,1%), HIV/AIDS (5,1%) e problemas de visão/cegueira (4,6%), e 18,7% diziam fazer uso de algum medicamento^(21)^.

Neste estudo, todos os entrevistados foram do sexo masculino, não sendo encontradas mulheres no local nos momentos das entrevistas. Apesar da ausência de informações quanto ao número de usuários dos sexos masculino e feminino atendidos pelo local, o resultado apresenta semelhança com outros estudos presentes na literatura e com o CadÚnico. Em relação à escolaridade, grande parte da PSR não chega a completar o ensino fundamental^(22)^. Porém, em relação à cor da pele, o presente trabalho difere dos dados contidos na literatura, visto que a maioria dos participantes se considera branca. Sobre o aspecto cor da pele, destaca-se que, conforme informações do último censo demográfico, o município de Botucatu apresenta população majoritariamente branca (69,6%), sendo que apenas 5,5% se autodeclaram negros, e 24%, pardos^(19)^.

Em relação à experiência de viver nas ruas, a PSR enfrenta diversas adversidades em seu cotidiano, gerando grande sobrecarga física e emocional. Dessa forma, “é andando que se conhece a rua, e é no processo de conhecê-la que se aprende por onde andar”^(23)^, e a partir disso, constrói-se um modo de existência, com maior predisposição para o adoecimento nas ruas^(24)^.

No que se refere ao sono, a PSR adormece mais durante o dia em decorrência do maior movimento das cidades, dando-lhe maior segurança para esse momento^(25)^. A noite geralmente é um período de maior exposição a risco para a PSR, acompanhada pelo uso de substâncias psicoativas e as baixas temperaturas climática que se intensificam durante a madrugada, fazendo da noite um período de alerta permanente^(25)^. Em outro estudo, a PSR relatou estar acostumada com essa situação^(26)^, narrativa semelhante de um dos entrevistados do presente trabalho. Isso pode estar associado à liberdade que a rua lhes dá, de poder utilizar as drogas e bebidas sem serem julgados por seus familiares.

Sentimentos como desconfiança, medo e indiferença são os mais retratados pela PSR, reforçando a invisibilidade social^(22)^. As “tecnologias de expulsão” e “arquitetura antimendigo” que a sociedade cria materializam cada vez mais a estigmatização e discriminação dessa população^(27)^.

A respeito da pandemia de COVID-19, a desigualdade social causa risco distinto de adoecimento. A necessidade de isolamento pôde criar barreiras no acolhimento em abrigos devido à demanda de espaço e materiais, e a permanência contínua de 14 dias. Além disso, há uma relação conflituosa entre a PSR e os abrigos, devido às regras rígidas quanto ao uso de álcool e drogas nesses locais, à proibição de famílias e casais permanecerem juntos e à entrada de animais de estimação^(6,22)^.

Quanto aos principais problemas e preocupações com a saúde, a literatura aponta que o uso de drogas e variáveis relacionadas à instabilidade e à ruptura familiar são os principais motivos dessa população ter ido para a rua, aspectos também presentes para a PSR deste estudo. Diversos autores destacam as mesmas dificuldades enfrentadas pelos entrevistados, acrescentando também o desemprego como uma das causas^(26)^. O rompimento amoroso ou familiar pode desencadear sentimentos de desilusão ou até mesmo depressão, podendo estar intimamente ligado ao uso de álcool e drogas^(22)^.

Além disso, os ambientes cultural e sociopolítico influenciam o uso e abuso de drogas lícitas e ilícitas^(28)^. Nas ruas, a droga acaba sendo um elemento de comunicação e interação social, em que há união de indivíduos com problemas em comum enfrentados nesse ambiente^(29)^. Soma-se a isso o custo de drogas como crack ou cachaça, que é inferior ao valor de uma refeição^(6)^. Isso impulsiona o uso desses produtos, além de acabarem mascarando a fome^(23)^ e o frio, sendo um meio para conseguir relaxar e dormir^(30)^.

As situações de violência, uso de álcool e outras drogas, e o não acompanhamento/tratamento de situações crônicas são os principais fatores para procura dos serviços de saúde^(26)^. Em relação aos problemas de saúde nesta população, os mais prevalentes são doenças crônicas não transmissíveis, uso abusivo de substâncias e infecções sexualmente transmissíveis, os quais também puderam ser visualizados na pesquisa com a presença de indivíduos com hipertensão arterial, diabetes mellitus, tuberculose e hepatite C^(25)^.

Em relação ao acesso e cuidado com a saúde dos participantes deste estudo, os resultados divergem dos achados da última pesquisa de abrangência nacional, ao constatar que, quanto ao acesso à saúde, 43,8% dos entrevistados afirmaram que procuram primeiro o hospital/emergência quando estão doentes, e 27,4% procuram alguma unidade de saúde da atenção primária^(21)^.

Os CnaRs atuam em diversos problemas e necessidades de saúde específicos para esta população, através de ações compartilhadas e integradas a Atenção Primária, formados por equipes multiprofissionais^(24)^. No entanto, devido aos diversos obstáculos que são postos a essa população, estes tendem a ter medo e desconfiança diante de uma abordagem profissional, por isso a importância da construção de vínculo e organização das equipes para que esses indivíduos tenham profissionais de referência^(25)^.

O CnaR também é responsável por realizar o cadastro da PSR, sendo o cartão SUS o documento mais estimado por essa população, visto que é feito e entregue na hora, sem a necessidade de comprovação de endereço ou apresentação de documentos preexistentes^(25)^.

A articulação da rede de saúde se faz necessária, pois possibilita ações conjuntas direcionadas à PSR, a fim de proporcionar um cuidado ampliado desses indivíduos e identificar suas necessidades, visto que, para essa população, o território de vida é amplo, dinâmico e móvel^(24,31)^.

A literatura científica também nos revela a compreensão sobre essa dicotomia com relação ao atendimento nos serviços de saúde, sendo a demora do atendimento o problema mais relatado, no entanto sempre é atendido. Os serviços mais procurados pela PSR são os serviços de emergência, isso porque essa população não costuma frequentar continuamente os serviços de saúde, fazendo essa comunicação apenas em momentos de emergências^(26)^.

Os serviços não estão preparados para atender às peculiaridades dessa população, pois ainda há muito preconceito e discriminação pelos próprios profissionais de saúde^(26)^. A falta de conhecimento dos profissionais e da sociedade acerca das experiências de vida da PSR reforça vulnerabilidades e a exclusão dessa população, contradizendo a carta constitucional e os princípios do SUS^(32)^.

Nesse contexto, salienta-se o perfil dos entrevistados, pessoas que vivem nas ruas, mas que se encontravam no momento da pesquisa em instituição de acolhimento e cuidado: Espaço Acolhedor. O usuário do Espaço Acolhedor se compromete a um conjunto de regras, como fazer a higiene pessoal e outras, além de contar com o apoio dos profissionais da instituição e da articulação intersetorial, aspectos que os diferenciam das pessoas que efetivamente estão vivendo nas ruas e que favorecem o acesso e o cuidado à saúde, sugerindo um viés e limitação da pesquisa.

### Limitações do estudo

Compreende-se que os achados aqui apresentados não esgotam a complexidade do fenômeno em análise. Faz-se necessário realizar outros estudos e recortes sobre o tema no intuito de continuarmos avançando na compreensão das várias determinações que atravessam e condicionam a problemática. Além disso, apesar de o estudo não planejar recorte de sexo para a participação, apenas pessoas do sexo masculino participaram das entrevistas. Essa limitação do estudo se deu em decorrência de não serem encontradas mulheres no local no momento da coleta de dados.

### Contribuições para a área da saúde, enfermagem ou políticas públicas

Reconhece-se que o trabalho desenvolvido revelou especificidades e particularidades sobre o fenômeno em análise, em condições de ampliar a compreensão sobre o tema e de suas inflexões para o cotidiano assistencial para enfermagem e demais áreas.

## CONSIDERAÇÕES FINAIS

Considera-se que o delineamento qualitativo permitiu uma compreensão da realidade social dos entrevistados e em suas produções humanas, particulares e construídas no mundo das relações e das representações, aspectos esses imensuráveis. Além disso, esse delineamento, que coaduna com as diretrizes curriculares nacionais do curso de medicina na formação de profissional médico generalista, humanista, crítico e reflexivo, representa uma alternativa ao modelo positivista e baseado no diálogo com as ciências humanas e ciências naturais, permitindo aos estudantes pesquisadores um diálogo com as ciências humanas e com as relações e intersubjetividade dos sujeitos entrevistados.

De modo geral, constatamos que as informações obtidas neste estudo corroboram os achados da literatura nacional e internacional sobre o tema, principalmente no que se refere às características sociodemográficas da PSR, com exceção do marcador cor da pele, o qual a maioria de nossos interlocutores se definiu enquanto homem branco.

A análise empregada por meio do método explicativo de Vygotsky nos permitiu compreender o fenômeno de modo particularizado e concatenado às idiossincrasias locais, com destaque para as Unidades Básicas de Saúde e equipe do CnaR do município, reconhecidos como principais agenciadores das demandas e necessidades de saúde dessa população.

O contexto da pandemia de COVID-19 não alterou significativamente o cotidiano desses indivíduos, mas é possível inferir que seus reflexos e desdobramentos também impactaram o fato de se encontrar e manter em situação de rua, colocando em evidência a complexidade e necessidade de ações intersetoriais e interprofissionais junto aos mesmos.

Reconhecemos as limitações de pesquisa com PSR desenvolvida a nível local, mas destacamos a importância de maiores estudos sobre suas experiências de acesso a serviços de saúde, em condições de contribuir para o aumento da visibilidade das principais demandas e necessidades de saúde dessa população, assim como na produção de informação que subsidie a elaboração e implantação de programas, políticas públicas e tomada de decisão em contextos semelhantes.

## DISPONIBILIDADE DE DADOS E MATERIAIS

Os dados de pesquisa só estão disponíveis mediante solicitação.
